# Effects of *Vaccinium*-derived antioxidants on human health: the past, present and future

**DOI:** 10.3389/fmolb.2024.1520661

**Published:** 2024-12-20

**Authors:** Amrita Ghosh, Samir C. Debnath, Abir U. Igamberdiev

**Affiliations:** ^1^ Department of Biology, Memorial University of Newfoundland, St. John’s, NL, Canada; ^2^ St. John’s Research and Development Centre, Agriculture and Agri-Food Canada, St. John’s, NL, Canada

**Keywords:** *Vaccinium*, berries, antioxidants, phenolics, anthocyanin, flavonoids

## Abstract

Dietary intake of *Vaccinium* berries has demonstrated significant potential in preventing many risk factors associated with metabolic syndromes in the human population. In recent years, a multitude of research has shown the role of antioxidants derived from *Vaccinium* berries on chronic diseases such as cardiovascular disorders, diabetes, obesity, and cancer. Several studies have also investigated the effect of *Vaccinium* berry consumption on their ability to modulate the risk factors associated with oxidative stress, vascular function, inflammation, and lipid metabolism. Regarding cancer, studies showed that the consumption of berries reduces inflammation, inhibits angiogenesis, protects against DNA damage within the cell, and controls apoptosis and proliferation rates in malignant tumours. However, which components are responsible for the health benefits is still unclear. Reports show that whole berry consumption usually confers positive effects on human health, and the health-promoting potentials are likely due to the presence of polyphenols with antioxidant activities. Among these polyphenols, various *Vaccinium* berry species have been reported to contain anthocyanins and flavonoids. These two polyphenolic compounds are known to have higher antioxidant activity and are beneficial for human health. There are now several studies and human clinical trials documenting the beneficial effects of *Vaccinium* berries, and these findings suggest that they may be promising for preventing and treating neurodegenerative diseases. This review focuses primarily on dietary *Vaccinium* berries consumption effects on human health and their potential role as therapeutic agents.

## 1 Introduction

The eternal pursuit of finding and identifying health-promoting agents has changed how we view our food sources. We have introduced superfoods, food supplements, and nutraceuticals in developed countries, reinforcing the food industry’s further growth (European Commission, 2006). Berries represent a large group of functional foods, also popularly known as “superfoods” due to their high content of disease-preventing and health-boosting chemicals (Ferlemi and Lamari, 2016). The genus *Vaccinium* L. (Ericaceae) includes approximately 450 diverse species, including these main commercial crops such as highbush blueberry (*V. corymbosum* L.), rabbiteye blueberry (*V. virgatum* Aiton, formerly known as *V. ashei* J.M.Reade), lowbush blueberry (*V. angustifolium* Aiton), bilberry (*V. myrtillus* L.), cranberry (*V. macrocarpon* Aiton), and lingonberry (*V. vitis-idaea* L.) (Retamales and Hancock, 2018). In general, berry fruit consumption has increased in recent years. Several research papers show that the increased consumption of berries is associated with a reduced risk of disorders linked with reactive oxygen species (ROS), such as cardiovascular disorders, cancer, and other inflammatory processes (Gomes-Rochette et al., 2016). A large number of scientific research cites the effect of berry consumption in three major groups: (a) physical and mental health maintenance; (b) reduction of the rate of obesity; and (c) decreased rate of chronic diet-related diseases, e.g., cardiovascular and metabolic disorders, type II diabetes (Giampieri et al., 2015). *Vaccinium* berries contain high concentrations of beneficial nutrients and other bioactive phytochemicals, which has led them to become the center of attraction for researchers working on the potential role of these phytochemicals in preventing chronic diseases (Colak et al., 2016). Many research papers have shown that these active compounds, which are present in phenolic form, are associated with high antioxidant activity. Additionally, there are several studies suggesting that wild *Vaccinium* Berries contain higher phenolic content and antioxidant activity than cultivated berries (Braga et al., 2013; Dinstel et al., 2013; Kanget al., 2015). Phenolic compounds present in *Vaccinium* berries are classified into diverse groups, which include phenolic acids such as hydroxybenzoic and hydroxycinnamic acids and their derivatives, flavonoids (flavonols, flavanols, and anthocyanins, and tannins. Tannins are further sub-grouped into condensed tannins like proanthocyanidins and hydrolysable tannins (Ferlemi and Lamari, 2016). Blueberries (*Vaccinium* spp.) contain the highest amount of *p*-coumaric acid, chlorogenic acid, and other caffeic acid derivatives, which are types of hydroxycinnamic acids (Mattila et al., 2006; Määttä-Riihinen et al., 2004a; Kylli, 2010; Häkkinen et al., 1999; Taruscio et al., 2004). When it comes to flavonoids among the *Vaccinium* berries, lingonberries (*V. vitis-idaea* L.), highbush blueberries, and American cranberries (*V. macrocarpon*) are known to be the richest source of flavonols such as quercetin and myricetin derivatives and aglycones (Määttä-Riihinen et al., 2004b; Määttä-Riihinen et al., 2004a; Kylli, 2010; Häkkinen et al., 1999; Taruscio et al., 2004). These chemical compounds possess high antioxidant activity (Wilms et al., 2005) and play a major role in preventing many chronic diseases (Castañeda-Ovando et al., 2009; Duthie et al., 2006). However, the concentration of these compounds depends on the species, genotype, growing condition and their post-harvesting techniques (Manganaris et al., 2014; Kresty et al., 2006).

Anthocyanins are important secondary plant metabolites, primarily occurring as glycosides of their aglycone anthocyanidins. The contents of the small edible berries are responsible for their bright colours as this pigment is evenly distributed in the epidermal tissues of the berries (Del Bo et al., 2015). The pigment in anthocyanins is water-soluble and responsible for orange, red, purple, and blue in fruits and vegetables (Delgado-Vargas et al., 2000). Anthocyanins are present in substantial quantities in glycosylated and various other forms in European cranberries (*V. oxycoccus*) and blueberries (Del Bo et al., 2015). European blueberries or bilberries contain fifteen anthocyanins, such as delphinidin and cyanidin monoglycosides, malvidin glycosides, petunidin, and peonidin. In the case of American cranberries, the principal anthocyanins are cyanidins, while in the case of European cranberries, it is peonidins (Norberto et al., 2013; Kaume et al., 2012; Gopalan et al., 2012; Seeram, 2008; Paredes-López et al., 2010). Similar to American cranberries, lingonberries also mainly contain cyanidin monoglycosides. Besides cyanidins, high and lowbush blueberries, lingonberries, and American cranberries contain procyanidins such as catechin and epicatechin polymers (Norberto et al., 2013; Zafra-Stone et al., 2007).

Commonly consumed *Vaccinium* berries have been studied for their effects on human health, but the nature and extent of their impact on humans remain vague. Hence, this article aims to provide a comprehensive overview of human clinical trials investigating the acute and chronic effects of *Vaccinium* berry polyphenols derived from fruits, their extracts and their derived products on inflammation, gut microbiota, diabetes, heart health, cancer, and brain activities.

## 2 Bioactive compounds

**FIGURE 1:**
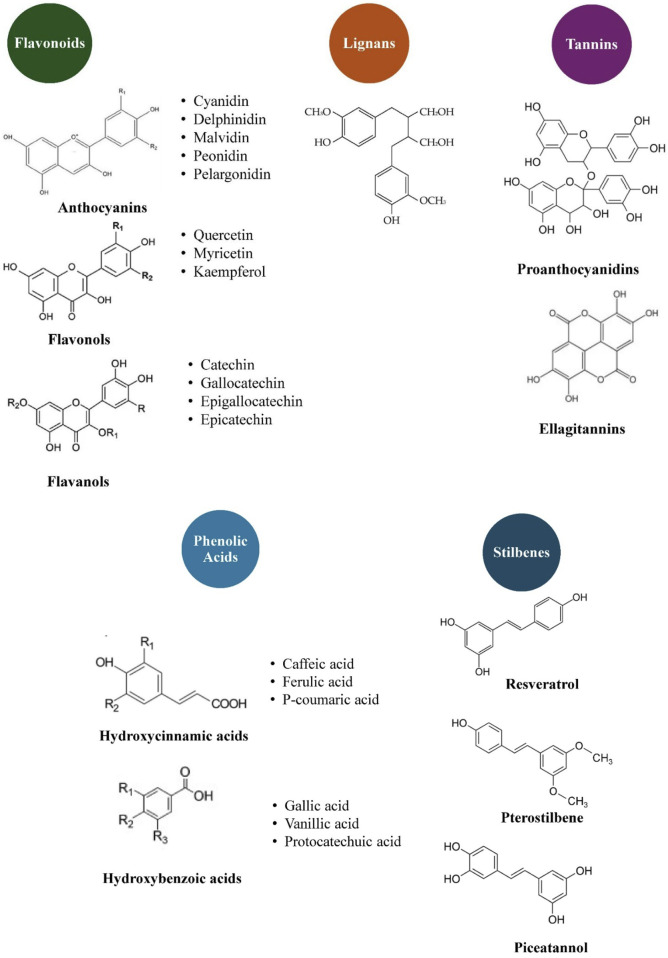
General classification of *Vaccinium* berry polyphenols and their main representatives.

Plants produce numerous bioactive compounds, which belong to different classes of secondary metabolites including polyphenols, phytosterols, lipoates, carotenoids, etc. (Acquaviva et al., 2021; He et al., 2024). In berries, the most abundant bioactive compounds are phenolics, which are mostly found in leaves, fruits, and seeds but can also be present in other parts of plants. The chemical structure of the phenolic compounds carries one or more aromatic rings with one or more hydroxyl groups (Szajdek and Borowska, 2008; Nile and Park, 2014; Del Bo et al., 2015; Skrovankova et al., 2015). Phenolics are either present in free or conjugated forms with water or fat-soluble compounds (Figure 1). Conjugated forms of phenolics are predominantly present as conjugated hydroxycinnamic acids, flavonol glycosides, and anthocyanins (Määttä-Riihinen et al., 2004a). These phenolics are not species-specific but shared across the genera. Among the berry polyphenols, anthocyanins constitute a large percentage. They have a characteristic C6−C3−C6 carbon structure and are glycosylated polyhydroxy and polymethoxy derivatives of flavylium salts (Wallace, 2011). A study has reported that anthocyanins have a glycosidic structure containing more than two sugar molecules, such as galactose, arabinose, xylose, and glucose, that effectively connect with aglycon and form through the phenylpropanoid pathway. Anthocyanins have more than 600 compounds and more than 30 anthocyanidin compounds (Bilawal et al., 2021). The major phenolic compounds (anthocyanins) found in *Vaccinium* berries are listed in Table 1. Anthocyanins are uniquely characterized by an oxonium ion on the C ring and are highly pigmented (Pandey and Rizvi, 2009). Among these anthocyanin compounds, quercetin, myricetin and their glycosidic derivatives reach up to 30%. Anthocyanins, including procyanidins and anthocyanidins such as cyanidin, malvidin, peonidin, delphinidin, and petunidin, can account for up to 24% of all polyphenolic compounds. Phenolic acids primarily include *p*-coumaric acid, chlorogenic acid, caffeic acid, ferulic acid and vanillic acid. They account for up to 12% of the total polyphenols (Nemzer et al., 2022).

**TABLE 1:** Major polyphenols found in *Vaccinium* berries.

Type of berry	Scientific name	Types of polyphenols	References
Bilberry	*Vaccinium myrtillus*	delphinidin-3-O-glucoside, cyanidin-3-O-glucoside, malvidin-3-O-glucoside	Määttä-Riihinen et al. (2004a)
Blueberry	*Vaccinium* spp.	delphinidin 3-O-galactoside, -arabinoside; malvidin 3-O-galactoside, petunidin 3-O-galactoside	Cho et al., 2004, Mäaättä-Riihinen et al., 2004a
Cranberry	*V. macrocarpon*	peonidin 3-O-galactoside, -arabinoside; cyanidin 3-O-galactoside	Zheng and Wang (2003)
Lingonberry	*V. vitis-idaea*	cyanidin 3-O-galactoside, peonidin 3-O-galactoside	Määttä-Riihinen et al. (2004a)

Plant phenolics were regarded as antinutritional and toxic for a long time as these compounds’ chemical nature of functioning as an inhibitor to proteolytic, lipolytic and glycolytic enzymes reduces their ability to absorb nutrients (Olas, 2018). However, the toxicity of the phenolic compounds derived from berries was generally unnoticed in the previous studies, while the benefits were observed. Apart from being a source of non-nutritive compounds such as phenolics (Singh and Basu, 2012), *Vaccinium* berries also contain a wide range of nutritive compounds such as simple sugars like glucose and fructose, minerals, for example, phosphorus, calcium, iron, potassium, magnesium, manganese, sodium and copper, etc. (Del Bo et al., 2015; Szajdek and Borowska, 2008). Iron and manganese are essential components of antioxidant enzymes among these above-mentioned minerals. Moreover, berries contain vitamins A and E, reducing inflammation and acting as antioxidants (Skrovankova et al., 2015). Apart from that, berries contain high concentrations of dietary fibres and low concentrations of lipids. These fibers reduce the concentration of low-density lipoproteins (LDL) in blood serum and reduce the chances of occurrence of cardiovascular and neurodegenerative diseases and cancer. All these nutritive, non-nutritive compounds, vitamins, and minerals in the *Vaccinium* berries synergistically affect human health (Olas et al., 2016).

Apart from the polyphenolic compounds, *Vaccinium* berries also contain major lipid groups such as unsaturated fatty acids, sterols, terpenoids, and others that have high biological activity. These lipids are different from those found in mammals, which is why consuming these lipids has a significant role in human metabolism (Klavins et al., 2015). The first report of the presence of lipids was investigated in cranberries (Croteau and Fagerson, 1969). Klavins et al. (2015) studied the lipid profile of blueberry, bilberry, lingonberry and cranberry grown in the wild in Latvian forests and bogs. The lipid profile revealed 111 different types of lipid fractions, including fatty acids, sterols, triterpenoids, alkanes, phenolic and carboxylic acids and carotenoids. Since then, this group of compounds has been studied in many berry species. However, there is no detailed report on lipids found in *Vaccinium* berries.

## 3 Biological activities

### 3.1 Overview of biological activities

Due to the response to the biotic and abiotic stresses, plants produce phytochemicals. They are also known as secondary metabolites. Like other fruits and vegetables, berries were found to be a great source of bioactive phytochemical components. Berry phytochemicals comprise bioactive components such as tannins, polysaccharides, alkaloids, vitamins, flavonoids, and other trace elements. They also contain sugar and fiber, which increases fruit taste and possesses many biological properties. In berries, these bioactive properties are directly related to the concentrations of the various phytochemicals in these fruits. Berry research has always been generally focused on their antioxidant properties. The antioxidant properties of these phytochemicals reduce oxidative damage to DNA, RNA, proteins and lipids at the cellular level by scavenging reactive oxygen species (ROS) (Bilawal et al., 2021). ROS are responsible for triggering aging and several inflammatory conditions, as well as cancer (Sosa et al., 2013; Nemzer et al., 2022). They also promote the regeneration of other antioxidants and endogenous antioxidant defense systems (Bujor et al., 2019). The imbalance between oxidants and antioxidants results in abnormalities. It produces significant ROS, including O_2_^−,^ HO^−^, NO, and RO^−^, which interferes with the cellular processes (Bujor et al., 2019; Maya-Cano et al., 2021). These superoxide ions can convert into hydrogen peroxide (H_2_O_2_), which can further convert into the highly reactive hydroxyl radical (OH^−^). Hydroxyl radicals, due to their high reactivity, cause oxidative damage, including lipid peroxidation in membranes, oxidative modification of proteins, and oxidative damage to DNA (Sosa et al., 2013).

**FIGURE 2:**
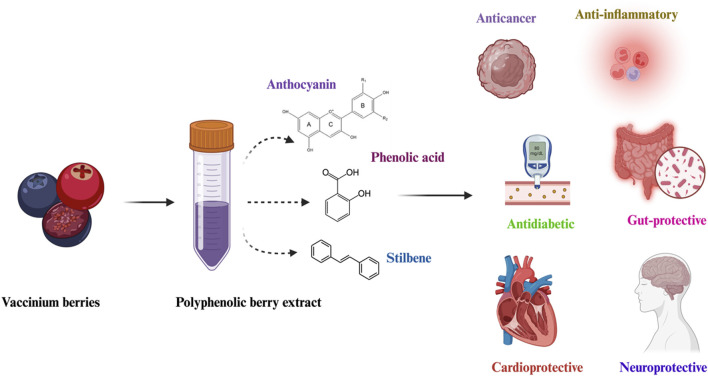
Bioactive properties of major *Vaccinium* berry-derived polyphenols.

Various methods have been used to determine the antioxidant activity of *Vaccinium* berries. Among them, the Folin-Ciocalteu method, the copper ion reducibility assay (CUPRAC), the ferric ion reducibility assay (FRAP), the DPPH (2, 2-diphenyl-1-picrylhydrazyl) radical scavenging method, and the ABTS method were mostly used in the scientific literature (Bujor et al., 2019). Goyali et al. (2013) examined the oxidative capacity of lowbush blueberry (*V. angustifolium*). It was found that the total phenolic content (TPC) value ranged from 34.2 to 42.7 mg GAE/g FW, total flavonoid content (TFC) from 12.7 to 22.3 mg CE/g FW, and proanthocyanidin content (PAC) from 4.7 to 6.5 mg CE/g FW in the greenhouse-grown and their cutting counterparts. In another study on half-high blueberries, results of the biochemical assays of the greenhouse-grown and somatic embryogenesis-derived plants revealed that TPC varied from 0.26 to 0.46 GAE/g lw, TFC varied from 7.93 to 11.65 CE/g lw, and antioxidant activity (AA) varied from 0.08 to 14.85 GAE/g lw. The results showed that the propagation method and genotype impact the phenols and flavonoids in the leaves (Ghosh et al., 2018). A study on *V. oxycoccos* and *V. macrocarpon* compared the AA by the Folin-Ciocalteu method. It was found that polyphenol quantity in *V. macrocarpon* was 296.3 mg/100 g fresh weight while in *V. oxycoccos*, it was 288.5 mg/100 g fresh weight. However, DPPH revealed that *V. oxycoccos* had a stronger antioxidant potential (16.4 μmol TE/g FW) than *V. macrocarpon* varieties (13.08 μmol TE/g FW), which led to the inference that the concentration of resveratrol in the analyzed samples of both the species that may have an impact on the AA of the varieties (Borowska et al., 2009). The antioxidant capacity of the *Vaccinium* species has been tested in *in-vivo* studies as well. A study was conducted on male *Drosophila melanogaster* to analyze the anti-aging effect of anthocyanins derived from the bilberry extracts. It was reported that the administration of anthocyanin extracts at the concentrations of 2.5, 5.0 and 10.0 mg/mL extended the life of the flies by 9.16%, 11.90% and 6.88%, respectively, compared to the control sample (Zhang and Dai, 2022). Several pieces of research show that the effect of phytochemicals derived from *Vaccinium* berries is directly associated with their anticancerous activities (Juranić and Žižak, 2005). Not only anticancerous properties, but numerous studies have shown that all these components are believed to hold a broad spectrum of biomedical functions, including anti-inflammatory, antimicrobial, antiviral and antioxidant properties (Figure 2) (Puupponen-Pimia et al., 2004; Seeram, 2008; Skrovankova et al., 2015). Due to their health-promoting activities, these berries are highly recommended for the human diet, as their antioxidant effects have been explored in several *in vitro* and *in vivo* studies (Juranić and Žižak, 2005; Skrovankova et al., 2015) (Table 2).

**TABLE 2:** Effect of *Vaccinium* berry consumption on metabolic syndrome and risk factors modulation.

Type of berry	Intervention	Results	References
Blueberry	8-week randomized (R), single bound (SB), placebo-controlled (PC), parallel intervention (PI)	Reduced systolic and diastolic blood pressure	Basu et al. (2010)
	Diabetic C57b1/6J mice model with acute feeding of blueberry extracts with Labrasol (Gavage- 500 mg/kg body wt.)	Lowered elevated blood glucose levels	Grace et al. (2009)
	8-week, R, double-blind (DB), PC, PI	Reduced systolic and diastolic blood pressure, increased NO plasma levels and superoxide dismutase activity	Johnson et al. (2015)
	*In vitro*-TC-tet model	2.8-fold increase in cell proliferation	Martineau et al. (2006)
	6-week R, DB, PC, crossover intervention (CI)	Reduced endogenous and oxidatively induced DNA damage	Riso et al. (2013)
	6-week R, DB, PC, PI	Higher insulin sensitivity	Stull et al. (2010)
	6-week R, DB, PC, PI	Increased blood pressure and insulin sensitivity	Stull et al. (2015)
Bilberry	4-week, R, C, PI	Decreased serum levels of CRP, IL-6, IL-15, TNF-α, MIG	Karlsen et al. (2010)
	8-week R, Controlled (C), PI	Reduced serum levels of hs-CRP, IL-6, IL-12 and inflammation score and decreased expression of MMD and CCR2 transcripts	Kolehmainen et al. (2012)
	5-week, R, CI	Decreased body weight, waist circumference, increased insulin sensitivity	Lehtonen et al. (2011)
	R, C, 2 arm, chronic feeding (24 weeks) in type II diabetic patients	Significantly lowered fasting plasma glucose and homeostasis model assessment for insulin resistance index	Li et al. (2015)
	Diabetic Mice model with chronic feeding of bilberry extract for 5 weeks	Improved hyperglycemia and insulin sensitivity	Takikawa et al. (2010)
Cranberry	8-week, R, DB, PC, PI	Reduced ox-LDL, MDA and HNE plasma/serum levels	Basu et al. (2011)
	12-week R, C, PI	Increased insulin levels after placebo treatment	Chambers and Camire (2003)
	Post-prandial (PP) 4-week R, DB, CI	Increased flow mediated dilation and reduced carotid-femoral pulse wave velocity (a measure of central aortic stiffness) and HDL-cholesterol	Dohadwala et al. (2011)
	12-week, R, PC, DB, PI	Lower total cholesterol	Lee et al. (2008)
	60 days, PI	Decreased serum homocysteine levels, lipoperoxidation, and protein oxidation, increased serum folic acid levels	Lozovoy et al. (2013)
	2-week intervention	Reduced BMI, plasma ox-LDL levels, and higher Total plasma antioxidant capacity	Ruel et al. (2005)
	12-week intervention (3x 4-week intervention with 125, 250, and 500 mL/day cranberry juice)	Decreased body weight, BMI, waist circumference, waist-to-hip ratio, total HDL cholesterol apo B, after intervention with 250 and 500 mL cranberry juice Increased plasma nitrite/nitrate following intervention with 500 mL, and higher plasma antioxidant capacity following 250- and 500-mL cranberry juice. Higher HDL cholesterol following 250 mL cranberry juice	Ruel et al. (2006)
	12-week intervention (3x 4-week intervention with 125, 250 and 500 mL/day cranberry juice)	Reduced ox-LDL following 250 and 500 mL cranberry juice and decreased systolic blood pressure, s-VCAM, ICAM plasma levels following 500 mL cranberry juice. Decreased ox-LDL, ICAM plasma levels in subjects With previous history of metabolic syndrome following 12-week intervention Higher HDL cholesterol following 250 and 500 mL cranberry juice	Ruel et al. (2008)
	4-week, PC, DB, CI	Reduced arterial stiffness and global blood pressure	Ruel et al. (2013)
	12-week, R, DB, PI	Reduced glucose level	Shidfar et al. (2012)
	PP intervention	Decreased plasma insulin and glycemic response	Wilson et al. (2008)
	PP cross-over Intervention	Decreased glycemic and insulinemic response following SDC-LS	Wilson et al. (2010)
Lingonberry	Administration of Quercetin-rich extract from lingonberry to C2C12 myoblasts	Increased insulin-independent glucose uptake and stimulated AMPK	Eid et al. (2010)
	RCT, 4 arm, PP crossover	Reduced sucrose-induced PP glucose and insulin concentrations during the first half an hour post-intake Prevented sucrose-induced late PP hypoglycemic response	Torronen et al. (2012)

Studies have reported that adding fruits to our diet reduces the risk of chronic diseases like cancer, type II diabetes, obesity, and cardiovascular disorders (Skrovankova et al., 2015). It was revealed that dietary intake of flavonoids is associated with a lowered risk of all-cause mortality (Liu et al., 2017), including CVD. However, it is important to understand that all subclasses of flavonoids are not equally involved with cardioprotection, as there is a huge gap between flavonoids’ bioavailability and bioactivity. Anthocyanins, a subclass of flavonoids, are one of the main phytochemicals present in *Vaccinium* berries. It was found that anthocyanins have a greater bioavailability than was previously estimated (Czank et al., 2013). Several *in-vitro* studies have shown that anthocyanins are as bioactive as their parent compounds and sometimes even more (Amin et al., 2015; Keane et al., 2016). Anthocyanins are known to stimulate anti-inflammatory, anti-atherogenic, antioxidant and vasodilatory actions (Castaneda-Ovando et al., 2009; Edwards et al., 2015; Wang et al., 1999). Not only that but there is also a growing body of evidence revealing that dietary inclusion of anthocyanins improves *in vivo* vascular health (Fairlie-Jones et al., 2017; Jennings et al., 2012) as they have a potential underlying mechanism of augmenting endothelial-derived nitric oxide (NO) bioavailability. It was found that anthocyanins can directly or indirectly increase NO availability either by upregulating endothelial nitric oxide synthase and L-arginine pathways or via optimizing nitrate-nitrite-NO pathway and reducing NO degradation by their antioxidant activities (Edwards et al., 2015; Rocha et al., 2014). NO is an important molecule as it regulates endothelial homeostasis. Anthocyanin displays strong antioxidant activities, and foods high in anthocyanin have been shown to improve endothelial function (Rodriguez-Mateos et al., 2013). Research on the effect of flavonoid intake on mortality showed that intake of anthocyanidins is positively correlated with decreased risk of CVD mortality (Summary relative risk = 0.89, 95% CI: 0.83, 0.95) when tested amongst 5 cohorts (Grosso et al., 2017). All the available research done *in vitro* and *in vivo* on antioxidant and anticancerous properties of *Vaccinium* berries has advanced our understanding of their effects on human health and diseases. Therefore, the current review comprehensively summarizes what is currently known about the medicinal potential of these berries.

### 3.2 Anti-inflammatory effect

Inflammation is known as the first line of defense in animals in response to the attack of pathogens, allergens, or any kind of tissue injury. As a result of the inflammatory response, macrophages, which are part of our immunity system, release inflammatory mediators such as interleukins, nitric oxide (NO), tumour necrosis factor-α (TNF-α), and prostaglandin (PGE2) (Joseph et al., 2014). Usually, overexpression of such mediators is associated with a response to type II diabetes, cancer, and cardiovascular diseases (CVDs) (Joseph et al., 2016). Over the years, much-accumulated data on pre-clinical studies of mice shows that *Vaccinium* berries, such as blueberries, reduce adiposity while increasing insulin sensitivity and decreasing inflammatory responses (Land Lail et al., 2021). A study on blueberries and blackberries disclosed that a daily dietary intake of 9–18.9 mg/kg BW of phenolic extract reduced cholesterol in blood plasma and metabolic dysfunctions induced by high-fat diet (HFD) in C57BL/6J mice (Joseph et al., 2016). Another study showed that the continuous intake of Nordic wild blueberries with HFD (45% fat) for 12 weeks slows down weight gain because of obesity-induced inflammation in C57BL/6 (Mykkänen et al., 2014). Similarly, HFD mixed with blueberry powder for 12 weeks helped to restore innate immune response and T-cell proliferation in HFD-induced obese mice (Lewis et al., 2018). This also shows that consuming an adequate quantity of blueberry with an HFD may target the crucial factors in immune response and inflammation. A study in macrophages (RAW 264.7) revealed that blueberry anthocyanin reduced the expressions of two cytokines, such as Tumor necrosis factor α (TNFα) and Interleukin 1β (IL-1β), after 3 h of treatment by suppressing the NF-kB pathway expression (Lee et al., 2014). A study on *V. floribundum* revealed that phenolic extract of the berries reduced lipid accumulation and inhibited the production of anti-inflammatory response-inducing enzymes such as PGE2, NO, COX-2, and iNOS in macrophages (LPS-RAW 264.7) (Schreckinger et al., 2010). The antioxidant and anti-inflammatory effects correlate with phenolic content in berries being dependent on the species, varieties, and geographical location (Grace et al., 2014).

### 3.3 Maintenance of gut microbiota and antimicrobial and antiviral activities

Dietary consumption of berries is also known to help grow bacteria in the gut. Berries contain polyphenols, which promote symbiotic bacteria, reducing dysbiosis disorders (Pap et al., 2021). Phenolics and flavonoids present in the berries are also found to be effective against pathogenic bacteria and fungi (Kranz et al., 2020). The human gut microbiota consists of viruses, bacteria, fungi, protozoa, and archaebacteria (Burgos-Edwards et al., 2020). A berry diet can promote the growth of beneficial microbiota and inhibit negative bacterial populations in the gut. Studies have shown that black raspberries and *Vaccinium* berries, such as blueberries and lingonberries, help increase the growth of *Lactobacillus* and some of their subspecies, the population of *Bifidobacterium*, and help in slowing down obesity-related problems (Pap et al., 2021). A study revealed that cranberry juice has a strong activity against co-adhesion and co-aggregation of oral plaque bacteria (Gupta et al., 2015). In another study, several types of berry extracts, including lingonberry (*V. vitis-idaea*), bilberry (*V. myrtillus*), were found to have antibacterial effects against commonly found pathogenic bacteria such as *Escherichia coli, Staphylococcus aureus, Listeria monocytogenes*, and *Bacillus cereus* (Tian et al., 2018). Berry and leaf extracts of lingonberry have shown maximum anti-microbial effects against *S. aureus* (strain ATCC-25923) and MRSA (clinical) oral cavity isolates (Kryvtsova et al., 2020). Research done on Romanian blueberry (var. Elliot) showed that minimum inhibitory concentration of leaf extract was discovered to be highly effective against bacterial strains such as *S. aureus, Escherichia faecalis, Rhodococcus equi, E. coli*, and *Klebsiella pneumoniae* and a few *Candida* fungal strains, such as *C. albicans, C. zeylanoides*, and *C. parapsilosis* (Ștefanescu et al., 2020).

### 3.4 Antidiabetic effect

Diabetes mellitus (DM) is a chronic disease which is associated with other lethal diseases, including hypertension, obesity, cardiovascular diseases, and hyperlipidemia. DM is classified into type I and type II; among these, type II contributes to more than 90% of all diabetes globally (Batool et al., 2021; Skyler et al., 2017). According to the World Health Organization (WHO), 108 million in 1980 and 422 million in 2014 were living with diabetes. A report done by Cho and co-workers in 2018 revealed that 451 million adults were diagnosed globally with diabetes, and it was predicted to reach up to 693 million by 2045 (Cho et al., 2018). Either form of DM is known to increase the risk of serious chronic illnesses such as blocking heart and blood vessels and affecting kidneys, eyes and nerve functions. This is due to the high blood sugar level, which affects and damages the nerves and the blood vessels controlling these organs. Blockage of the heart and blood vessels may also cause complications like CAD and stroke (Edirisinghe and Burton-Freeman, 2016). Not only CAD but CVD is also known to be the prime cause of death in patients with DM (Grundy et al., 1999). Several researchers have investigated and supported the idea that berry polyphenols have an antidiabetic effect, which is usually associated with glucose homeostasis. Glucose homeostasis can be regulated in an insulin-dependent and independent manner. Polyphenols derived from berries have been studied for years for their effects on insulin-dependent glucose metabolism. This can be achieved by regulating insulin secretion via modulating pancreatic-cell function and peripheral tissue sensitivity (Edirisinghe and Burton-Freeman, 2016). It was found that Canadian blueberry extracts increased 3H-thymidine incorporation in TC-tet cells and increased cell proliferation by 2.8-fold (Martineau et al., 2006). In another study, it was seen that dietary supplementation of freeze-dried whole blueberry powder in a double-blinded and placebo-controlled sensitivity had antidiabetic effects in obese, nondiabetic, and insulin-resistant human participants (p < 0.05) when administered for over 6 weeks and reported to improve insulin sensitivity (Stull et al., 2010). In diabetic C57b1/6J mice, feeding them a blueberry diet also displayed antidiabetic activity. Blueberry fraction enriched with phenolics and anthocyanin, in addition to Labrasol (a pharmaceutically acceptable self-micro emulsifying drug delivery system), was reported to lower raised blood glucose levels when fed to diabetic C57b1/6J mice. The hypoglycemic effect of the concoction was equivalent to that of metformin, a well-known antidiabetic drug (Grace et al., 2009). A study on postprandial healthy women showed that the administration of either whole lingonberries or extracts reduced sucrose-induced postprandial glucose and insulin concentrations throughout the first 30 min of consumption. Furthermore, it was seen that during the second-hour post-intake, the concentration declined slowly but improved the overall glycemic profile (p < 0.05). Additionally, the investigation showed that whole berries and extracts stopped the sucrose-induced late postprandial hypoglycemic response and the compensatory free fatty acid recovery (Torronen et al., 2012). Schell et al. (2017) experimented with the anti-diabetic activities of cranberry extract in T2D and revealed that administration of dried cranberry significantly improved the postprandial glucose excursion. These situations revealed that the increasing incidence of DM and its associated diseases can be controlled with a *Vaccinium* berry-rich diet.

### 3.5 Cardioprotective effect

Several studies have shown a strong connection between berry-derived anthocyanins and cardiovascular health. Clinical studies such as the Kuopio Ischemic Heart Disease Risk Factor Study for a follow-up of around 13 years revealed a considerably lowered risk of CVD-associated death among men who had a significantly higher quartile of berry intake (>408 g/day) than men with the lowest intake (<133 g/day) (Basu et al., 2010). Although these results positively impacted CVD risk factors, the models also showed an inverse correlation between the intake of fruits, berries, and vegetables and serum haptoglobin in blood, an inflammation marker (Rissanen et al., 2003). A large group of postmenopausal women (n = 34,489) contributing to a CVD mortality study associated with blueberry intake for a 16-year follow-up period at Iowa Women’s Health Study found that consumption of blueberries once a week significantly decreased coronary heart disease mortality using an age-and energy-adjusted model (Mink et al., 2007). The most significant conclusion drawn from all these clinical studies was that dietary inclusion of berries in everyday diet might decrease LDL oxidation and lipid peroxidation, reduce plasma glucose or total cholesterol, and increase HDL cholesterol and plasma or urinary antioxidant capacity (Basu et al., 2010). As a high content of plasma glucose, lipids, and lipid oxidation have been associated with coronary artery disease (CAD), these researchers suggested that edible berries, including berries from the *Vaccinium* family, can be consumed to reduce the risk factors of CAD significantly (Krentz, 2003; Gupta et al., 2009). It was further shown that the regular inclusion of berries in the diet also reduces the postprandial metabolic and oxidative stresses, which are also associated with CAD (O’Keefe et al., 2008). Cranberry has been proven to be very effective against several health issues, including the management of systolic blood pressure in healthy men (Ruel et al., 2008). Another study showed that cranberry extract positively affects lipid profiles in subjects with type I or II DM (Lee et al., 2008). Various other studies showed that consuming blueberries and cranberries significantly decreases postprandial oxidative stress, specifically lipid peroxidation (Pedersen et al., 2000; Kay and Holub, 2002; Mazza et al., 2002; Ruel et al., 2005; Ruel et al., 2006). Many studies suggest the inverse correlation of flavonoid (specifically anthocyanin) intake with the occurrence of CVD and the elevated risk factors involved with CVD (Cassidy et al., 2011; Jennings et al., 2012; McCullough et al., 2012). Berry polyphenols positively affect the lipid profile and endothelial function of the blood vessels by reducing blood pressure and platelet accumulation (Pojer et al., 2013; Rodriguez-Mateos et al., 2014a; Rodriguez-Mateos et al., 2014c). Not only that, but their antioxidant and anti-inflammatory activities also support cardiovascular health (Rodriguez-Mateos et al., 2014a; Pojer et al., 2013). Not only the berry extracts from fresh or frozen fruits but baked goods with lowbush blueberries (*V. angustifolium*) exhibited similar effects on endothelial function (FMV) as with the drink made with freeze-dried blueberry powder (Rodriguez-Mateos et al., 2014b). All this available research suggests that dietary inclusion of berries can potentially be used as a therapy for pre-hypertension and hypertension management (Basu et al., 2010). However, none of these clinical trials were found to interfere with biomarkers responsible for inflammation, except in one case, it was found that cranberry juice supplementation substantially decreases adhesion molecules in healthy volunteers (Ruel et al., 2008).

### 3.6 Anticancerous effect

Among all the other healthy eating habits, including berries in the everyday diet is one of the most promising ways to prevent cancer (Baby et al., 2018). Phytochemicals present in the berry extracts influence genome stability at several stages, such as malignant transformation, initiation modulation, promotion and progression of cancer (Duthie, 2007). In general, berry extracts combat carcinogenesis in animal models. However, when exposed to chemical carcinogens, blueberry extracts did not protect animal models. DNA damage was noticed in the tumours, and there was no evidence of a reduction in the proliferation rate of the cancerous cells or size of the tumours when pre- or co-treated with blueberry extract. These results further suggested that despite having higher antioxidant capacity than other berry species, blueberries are deficient in one or more cryoprotective phytochemicals, preventing chemically induced cancer in the animal model (Aziz et al., 2002).

It was reported that the growth of the HT-29 cell lines in colon cancer was significantly inhibited using phenolics, such as anthocyanins and flavonols extracted from cranberry juice (Desrouillères et al., 2020). In another study, lingonberry-derived quercetin and procyanidin-A2 displayed anticancerous activity against colon (HT-29), melanoma (IGR39), and renal (CaKi-1) cancer. It was also observed that quercetin demonstrated the best anticancerous activity against renal cell carcinoma (CaKi-1) (Vilkickyte et al., 2020). Fermented catechol extracted from fermented Rabbiteye blueberry (*V. virgatum*) extract along with *Lactiplantibacillus plantarum* (CK10) resulted in inducing apoptosis and inhibiting HeLa cell multiplication after 24–72 h of administration (Ryu et al., 2019). Various flavonol compounds such as kaempferol, quercetin, and genistein acid extracted from bilberry or European blueberry (*V. myrtillus*) demonstrated cytotoxic effects against HCT-116 colon cancer cells. This study further showed that kaempferol had better anticancerous activity than other flavonols, inducing apoptosis by preventing apoptosis proteins (IAPs) inhibitors (Sezer et al., 2019). Extracts from Vaccinium-berries such as blueberry, bilberry, cranberry, and lingonberry contain anthocyanins and ellagic acid, which are known to exhibit anticarcinogenic activities (Seeram, 2008). Other reports suggest that cranberry extracts and press cake can significantly inhibit cell growth in breast, prostate, skin, brain and liver cancer cases by stopping the G1 stage of the cell cycle and initiating apoptosis (Sun et al., 2002; Sun and Liu, 2006). Additionally, bilberry extracts were found to induce programmed cell death in patients with leukemia (Katsube et al., 2003). Extracts of several fruits, including blueberries, blackcurrant, black chokeberries, and raspberries, showed a strong antagonistic effect on the proliferation of breast cancer cell line MCF-7 and the colon cancer cell line HT29 and reduced their growth by up to 74% (Olsson et al., 2004).

### 3.7 Neuroprotective activity

There is much evidence supporting that oxidative stress of reactive oxygen species (ROS) in cells is responsible for the progression of neurodegenerative diseases such as Parkinson’s disease, Huntington’s disease, amyotrophic lateral sclerosis, and Alzheimer’s disease (Lim et al., 2024), and berry-derived antioxidants were effective against neurodegenerative diseases (Nile and Park, 2014). Berry antioxidants also demonstrate neuroprotective activities, and several studies have shown that phenolic components from the *Vaccinium* species have anti-inflammatory and neuroprotective effects. In a study done with blueberry and lingonberry, brain-derived cell cultures from rats were found to be significantly tolerant against glutamate excitotoxicity when treated with blueberry extracts for 24 h. However, lingonberry (*V. vitis-idaea* L.) extracts failed to provide any protection against it. Additionally, leaf extracts of blueberry and lingonberry displayed significant neuroprotective effects, while among the fruits, only blueberry fruits showed neuroprotection on the same brain cells (Vyas et al., 2013). That is why further work has been done to investigate the neuroprotective activity of blueberry leaf extract in microglial cells derived from mice. Microglial cells are the brain’s first line of defence cells; glutamate or α-synuclein was administered to microglial cells to induce an inflammatory response. The cells were treated with blueberry fruit and leaf extracts, which decreased cell death and reduced inflammation after 24 h. This result further points to the fact that a blueberry-rich diet, with leaves or fruits, can protect against neurodegenerative disorders (Debnath-Canning et al., 2020). Over the years, studies have suggested that the inclusion of blueberries in a regular diet may help with age-related and oxidative stress, which are responsible for declined brain function (Wang et al., 2005; Krikorian et al., 2010). Another report suggested that a blueberry-supplemented diet can improve behavioural deficits associated with age or a high-fat diet (Carey et al., 2014). Overproducing ROS and reactive nitrogen species (RNS) free radicals cause aging and neurodegenerative diseases. Cortical cell cultures derived from neonatal rat pups were intoxicated with glutamate for 24 h, and it was seen that glutamate was responsible for morphological disruptions such as increased formation of dark punctae and disruption of cell bodies. Glutamate-treated cells were administered with lingonberry and blueberry leaf and fruit extract. They were found that, while lingonberry fruits failed to provide any protection from glutamate toxicity, the leaf extracts from both the berries and blueberry fruit extract displayed no cell death in the presence of glutamate (Kalidindi, 2014).

It was found that aged rats fed with blueberries have shown a reduction of ischemia-induced apoptosis in brain cells which is due to their capability of interacting with ROS and RNS, which accumulated during the ischemic phase in the central nervous system (Wang et al., 2005; Andres-Lacueva et al., 2005). Krikorian et al. (2010) reported in older humans that improved memory capabilities were detected by increased synaptic plasticity as a result of microglial modulation of the microglia-neuron crosstalk through the increase of the expression of CX3CR1 receptor was associated with a blueberry rich diet (Meireles et al., 2016). Memory loss is often associated with oxidative damage to lipids, proteins, and nucleic acids. Oxidative damage to all three can disrupt neural function. It was seen that bilberry extract was significantly effective against oxidative damage by decreasing lipid peroxides and increasing superoxide dismutase activity in the brain. Additionally, it was found that long-term supplementation of bilberry extract in the diet of the OXYS rats prevented learning and memory deficits (Rahman, 2007; Uttara et al., 2009). These findings specifically signify the effect of *Vaccinium* berry antioxidants on the neuroprotection of brain function.

## 4 Conclusion

Several *in vitro* and *in vivo* studies now indicate that berries positively impact human health by acting as strong anticancer and antioxidant agents. They are an ideal dietary source of bioactive components and could play a role in reducing cancer risk. The unique phytochemical constituents in berries act individually or synergistically to provide protection against several health disorders, including cancer and CADs. It is evident from this review that a lot has been done in this direction, but much more needs to be done to pinpoint the molecular mechanisms associated with the most beneficial phytochemicals that make up these nutritious fruits. The review summarizes the effects of bioactive compounds present in *Vaccinium* berries and their function against cardiovascular and neurodegenerative diseases. It was seen that measurable criteria like total anthocyanin or total phenolic content and total antioxidant content may also be associated with the effectiveness of health benefits. Overall, more *in vivo* data are required to understand the mechanisms of action, while more human clinical trials using different parameters such as gender, age, and any pre-existing condition should be performed such new information on the bioactive components of berries can be revealed, and the existing information could be validated. Also, using berry phenolic compounds as antimicrobial agents provides many possibilities for use in the food and medical industry. It will also be a very interesting topic for future research priority by developing new ways for berry compounds to avoid and manage antibiotic-resistant infections. In addition to the phenolic compounds, phytosterols are well-known for their antioxidant activities. Epidemiological and experimental reports suggest that they help reduce cholesterol and potentially protect against several types of cancer. Furthermore, berry lipids are also used in many commercial products. These ignited a general interest in studying these compounds in depth to understand their potential application in cosmetics, pharmacy and the food industry.

## References

[B1] Acquaviva R. Malfa G. A. Di Giacomo C. (2021). Plant-based bioactive molecules in improving health and preventing lifestyle diseases. Int. J. Mol. Sci. 22 (6), 2991. 10.3390/ijms22062991 33804225 PMC8000372

[B2] Amin H. P. Czank C. Raheem S. Zhang Q. Botting N. P. Cassidy A. (2015). Anthocyanins and their physiologically relevant metabolites alter the expression of IL-6 and VCAM-1 in CD40L and oxidized LDL challenged vascular endothelial cells. Mol. Nutr. and Food Res. 59, 1095–1106. 10.1002/mnfr.201400803 25787755 PMC4950056

[B3] Andres-Lacueva C. Shukitt-Hale B. Galli R. L. Jauregui O. Lamuela-Raventos R. M. Joseph J. A. (2005). Anthocyanins in aged blueberry-fed rats are found centrally and may enhance memory. Nutr. Neurosci. 8 (2), 111–120. 10.1080/10284150500078117 16053243

[B4] Aziz R. M. Nines R. Rodrigo K. Harris K. Hudson T. Gupta A. (2002). The effect of freeze-dried blueberries on N-nitrosomethylbenzylamine tumorigenesis in the rat esophagus. Health Environ. Res. Online 40, 43–49. 10.1076/phbi.40.7.43.9174

[B5] Baby B. Antony P. Vijayan R. (2018). Antioxidant and anticancer properties of berries. Crit. Rev. Food Sci. Nutr. 58 (15), 2491–2507. 10.1080/10408398.2017.1329198 28609132

[B6] Basu A. Betts N. M. Ortiz J. Simmons B. Wu M. Lyons T. J. (2011). Low-energy cranberry juice decreases lipid oxidation and increases plasma antioxidant capacity in women with metabolic syndrome. Nutr. Res. 31 (3), 190–196. 10.1016/j.nutres.2011.02.003 21481712 PMC3075541

[B7] Basu A. Rhone M. Lyons T. J. (2010). Berries: emerging impact on cardiovascular health. Nutr. Rev. 68 (3), 168–177. 10.1111/j.1753-4887.2010.00273.x 20384847 PMC3068482

[B8] Batool S. R. Hasan I. Dars J. A. Batool R. Ahmed S. M. Iqbal M. (2021). Assessment of sleep quality in patients with type 2 diabetes mellitus: a prospective observational study from a tertiary care centre. Pak. Armed Forces Med. J. 71 (6), 1954–1957. 10.51253/pafmj.v6i6.4585

[B9] Bilawal A. Ishfaq M. Gantumur M.-A. Qayum A. Shi R. Fazilani S. A. (2021). A review of the bioactive ingredients of berries and their applications in curing diseases. Food Biosci. 44, 101407. 10.1016/j.fbio.2021.101407

[B10] Borowska E. J. Mazur B. Kopciuch R. G. Buszewski B. (2009). Polyphenol, anthocyanin and resveratrol mass fractions and antioxidant properties of cranberry cultivars. Food Technol. Biotechnol. 47 (1), 56–61.

[B11] Braga P. C. Antonacci R. Wang Y. Y. Lattuada N. Dal Sasso M. Marabini L. (2013). Comparative antioxidant activity of cultivated and wild Vaccinium species investigated by EPR, human neutrophil burst and COMET assay. Eur. Rev. Med. Pharmacol. Sci. 17 (15), 1987–1999. 23884818

[B12] Bujor O. C. Tanase C. Popa M. E. (2019). Phenolic antioxidants in aerial parts of wild Vaccinium species: towards pharmaceutical and biological properties. Antioxidants 8 (12), 649. 10.3390/antiox8120649 31888242 PMC6943522

[B13] Burgos-Edwards A. Fernández-Romero A. Carmona M. Thuissard-Vasallo I. Schmeda-Hirschmann G. Larrosa M. (2020). Effects of gastrointestinal digested polyphenolic enriched extracts of Chilean currants (*Ribes magellanicum* and *Ribes punctatum*) on *in vitro* fecal microbiota. Food Res. Int. 129, 108848. 10.1016/j.foodres.2019.108848 32036928

[B14] Carey A. N. Gomes S. M. Shukitt-Hale B. (2014). Blueberry supplementation improves memory in middle-aged mice fed a high-fat diet. J. Agric. Food Chem. 62 (18), 3972–3978. 10.1021/jf404565s 24446769

[B15] Cassidy A. O’Reilly É. J. Kay C. Sampson L. Franz M. Forman J. P. (2011). Habitual intake of flavonoid subclasses and incident hypertension in adults. Am. J. Clin. Nutr. 93 (2), 338–347. 10.3945/ajcn.110.006783 21106916 PMC3021426

[B16] Castañeda-Ovando A. de Lourdes Pacheco-Hernández M. Páez-Hernández M. E. Rodríguez J. A. Galán-Vidal C. A. (2009). Chemical studies of anthocyanins: a review. Food Chem. 113 (4), 859–871. 10.1016/j.foodchem.2008.09.001

[B17] Chambers B. K. Camire M. E. (2003). Can cranberry supplementation benefit adults with type 2 diabetes? Diabetes Care 26 (9), 2695–2696. 10.2337/diacare.26.9.2695-a 12941742

[B18] Cho M. J. Howard L. R. Prior R. L. Clark J. R. (2004). Flavonoid glycosides and antioxidant capacity of various blackberry, blueberry and red grape genotypes determined by high‐performance liquid chromatography/mass spectrometry. J. Sci. Food Agric. 84 (13), 1771–1782. 10.1002/jsfa.1885

[B19] Cho N. H. Shaw J. E. Karuranga S. Huang Y. da Rocha Fernandes J. D. Ohlrogge A. (2018). IDF Diabetes Atlas: global estimates of diabetes prevalence for 2017 and projections for 2045. Diabetes Res. Clin. Pract. 138, 271–281. 10.1016/j.diabres.2018.02.023 29496507

[B20] Colak N. Torun H. Gruz J. Strnad M. Subrtova M. Inceer h. (2016). Comparison of phenolics and phenolic acid profiles in conjunction with oxygen radical absorbing capacity (ORAC) in berries of *Vaccinium arctostaphylos* L. and *V. myrtillus* L. Pol. J. Food Nutr. Sci. 66 (2), 85–91. 10.1515/pjfns-2015-0053

[B21] Croteau R. Fagerson I. S. (1969). Seed lipids of the American cranberry (*Vaccinium macrocarpon*). Phytochemistry 8, 2219–2222. 10.1016/s0031-9422(00)88185-6

[B22] Czank C. Cassidy A. Zhang Q. Morrison D. J. Preston T. Kroon P. A. (2013). Human metabolism and elimination of the anthocyanin, cyanidin-3-glucoside: a 13C-tracer study. Am. Clin. Nutr. 97, 995–1003. 10.3945/ajcn.112.049247 23604435

[B23] Debnath-Canning M. Unruh S. Vyas P. Daneshtalab N. Igamberdiev A. U. Weber J. T. (2020). Fruits and leaves from wild blueberry plants contain diverse polyphenols and decrease neuroinflammatory responses in microglia. J. Funct. Foods 68, 103906. 10.1016/j.jff.2020.103906

[B24] Del Bo C. Martini D. Porrini M. Klimis-Zacas D. Riso P. (2015). Berries and oxidative stress markers: an overview of human intervention studies. Food and Funct. 6 (9), 2890–2917. 10.1039/C5FO00657K 26226324

[B25] Delgado-Vargas F. Jiménez A. Paredes-López O. (2000). Natural pigments: carotenoids, anthocyanins, and betalains—characteristics, biosynthesis, processing, and stability. Crit. Rev. Food Sci. Nutr. 40 (3), 173–289. 10.1080/10408690091189257 10850526

[B27] Desrouillères K. Millette M. Bagheri L. Maherani B. Jamshidian M. Lacroix M. (2020). The synergistic effect of cell wall extracted from probiotic biomass containing *Lactobacillus acidophilus* CL1285, L. casei LBC80R, and *L. rhamnosus* CLR2 on the anticancer activity of cranberry juice—HPLC fractions. J. Food Biochem. 44 (5), e13195. 10.1111/jfbc.13195 32185816

[B107] Dinstel R. Cascio J. Koukel S. (2013). The antioxidant level of Alaska's wild berries: high, higher and highest. Int. J. Circumpolar Health 72 (1), 21188. 10.3402/ijch.v72i0.21188 PMC3751288 23977647

[B28] Dohadwala M. M. Holbrook M. Hamburg N. M. Shenouda S. M. Chung W. B. Titas M. (2011). Effects of cranberry juice consumption on vascular function in patients with coronary artery disease. Am. J. Clin. Nutr. 93 (5), 934–940. 10.3945/ajcn.110.004242 21411615 PMC3076649

[B29] Duthie S. J. (2007). Berry phytochemicals, genomic stability and cancer: evidence for chemoprotection at several stages in the carcinogenic process. Mol. Nutr. and Food Res. 51 (6), 665–674. 10.1002/mnfr.200600257 17487926

[B30] Duthie S. J. Jenkinson A. M. Crozier A. Mullen W. Pirie L. Kyle J. (2006). The effects of cranberry juice consumption on antioxidant status and biomarkers relating to heart disease and cancer in healthy human volunteers. Eur. J. Nutr. 45, 113–122. 10.1007/s00394-005-0572-9 16032375

[B31] Edirisinghe I. Burton-Freeman B. (2016). Anti-diabetic actions of Berry polyphenols–Review on proposed mechanisms of action. J. Berry Res. 6 (2), 237–250. 10.3233/JBR-160137

[B32] Edwards M. Czank C. Woodward G. M. Cassidy A. Kay C. D. (2015). Phenolic metabolites of anthocyanins modulate mechanisms of endothelial function. J. Agric. Food Chem. 63, 2423–2431. 10.1021/jf5041993 25686009

[B33] Eid H. M. Martineau L. C. Saleem A. Muhammad A. Vallerand D. Benhaddou-Andaloussi A. (2010). Stimulation of AMP-activated protein kinase and enhancement of basal glucose uptake in muscle cells by quercetin and quercetin glycosides, active principles of the anti-diabetic medicinal plant *Vaccinium vitis-idaea* . Mol. Nutr. Food Res. 54 (7), 991–1003. 10.1002/mnfr.200900218 20087853

[B148] European Commission (2006). Regulation No. 1924/2006 on nutrition and health claims made on foods. Official Journal of the European Union L12/3–L12/18.

[B34] Fairlie-Jones L. Davison K. Fromentin E. Hill A. M. (2017). The effect of anthocyanin-rich foods or extracts on vascular function in adults: a systematic review and meta-analysis of randomised controlled trials. Nutrients 9, 908. 10.3390/nu9080908 28825651 PMC5579701

[B35] Ferlemi A.-V. Lamari F. N. (2016). Berry leaves: an alternative source of bioactive natural products of nutritional and medicinal value. Antioxidants 5 (2), 17. 10.3390/antiox5020017 27258314 PMC4931538

[B36] Ghosh A. Igamberdiev A. U. Debnath S. C. (2018). Thidiazuron-induced somatic embryogenesis and changes of antioxidant properties in tissue cultures of half-high blueberry plants. Sci. Rep. 8 (1), 16978. 10.1038/s41598-018-35233-6 30451961 PMC6242952

[B37] Giampieri F. Forbes-Hernandez T. Y. Gasparrini M. Alvarez-Suarez J. M. Afrin S. Bompadre S. (2015). Strawberry as a health promoter: an evidence based review. Food and Funct. 6 (5), 1386–1398. 10.1039/C5FO00147A 25803191

[B38] Gomes-Rochette F. Da Silveira Vasconcelos M. M. Nabavi S. F. Mota E. Cs Nunes-Pinheiro D. Daglia M. (2016). Fruit as potent natural antioxidants and their biological effects. Curr. Pharm. Biotechnol. 17 (11), 986–993. 10.2174/1389201017666160425115401 27109905

[B39] Gopalan A. Reuben S. C. Ahmed S. Darvesh A. S. Hohmann J. Bishayee A. (2012). The health benefits of blackcurrants. Food and Funct. 3 (8), 795–809. 10.1039/C2FO30058C 22673662

[B40] Goyali J. C. Igamberdiev A. U. Debnath S. C. (2013). Morphology, phenolic content and antioxidant capacity of lowbush blueberry (*Vaccinium angustifolium* Ait.) plants as affected by *in vitro* and *ex vitro* propagation methods. Can. J. Plant Sci. 93 (6), 1001–1008. 10.4141/cjps2012-307

[B41] Grace M. H. Esposito D. Dunlap K. L. Lila M. A. (2014). Comparative analysis of phenolic content and profile, antioxidant capacity, and anti-inflammatory bioactivity in wild Alaskan and commercial Vaccinium berries. J. Agric. Food Chem. 62 (18), 4007–4017. 10.1021/jf403810y 24219831 PMC4026347

[B42] Grace M. H. Ribnicky D. M. Kuhn P. Poulev A. Logendra S. Yousef G. G. (2009). Hypoglycemic activity of a novel anthocyanin-rich formulation from lowbush blueberry, *Vaccinium angustifolium* Aiton. Phytomedicine 16 (5), 406–415. 10.1016/j.phymed.2009.02.018 19303751 PMC2718544

[B43] Grosso G. Micek A. Godos J. Pajak A. Sciacca S. Galvano F. (2017). Dietary flavonoid and lignan intake and mortality in prospective cohort studies: systematic review and dose-response meta-analysis. Am. J. Epidemiol. 185, 1304–1316. 10.1093/aje/kww207 28472215

[B44] Grundy S. M. Benjamin I. J. Burke G. L. Chait A. Eckel R. H. Howard B. V. (1999). Diabetes and cardiovascular disease: a statement for healthcare professionals from the American Heart Association. Circulation 100 (10), 1134–1146. 10.1161/01.CIR.100.10.1134 10477542

[B45] Gupta A. Bansal K. Marwaha M. (2015). Effect of high-molecular-weight component of Cranberry on plaque and salivary Streptococcus mutans counts in children: an: *in vivo*: study. J. Indian Soc. Pedod. Prev. Dent. 33 (2), 128–133. 10.4103/0970-4388.155125 25872631

[B46] Gupta S. Sodhi S. Mahajan V. (2009). Correlation of antioxidants with lipid peroxidation and lipid profile in patients suffering from coronary artery disease. Expert Opin. Ther. targets 13 (8), 889–894. 10.1517/14728220903099668 19606928

[B47] Häkkinen S. Heinonen M. Kärenlampi S. Mykkänen H. Ruuskanen J. Törrönen R. (1999). Screening of selected flavonoids and phenolic acids in 19 berries. Food Res. Int. 32 (5), 345–353. 10.1016/S0963-9969(99)00095-2

[B48] He W. S. Zhao L. Sui J. Li X. Huang S. Ding H. (2024). Enzymatic synthesis of a novel antioxidant octacosanol lipoate and its antioxidant potency in sunflower oil. J. Agric. Food Chem. 72 (39), 21781–21793. 10.1021/acs.jafc.4c07240 39289871 PMC11450929

[B49] Jennings A. Welch A. A. Fairweather-Tait S. J. Kay C. Minihane A. M. Chowienczyk P. (2012). Higher anthocyanin intake is associated with lower arterial stiffness and central blood pressure in women. Am. J. Clin. Nutr. 96 (4), 781–788. 10.3945/ajcn.112.042036 22914551

[B149] Johnson S. A. Figueroa A. Navaei N. Wong A. Kalfon R. Ormsbee L. K. (2015). Daily blueberry consumption improves blood pressure and arterial stiffness in postmenopausal women with pre- and stage 1-hypertension: a randomized, double-blind, placebo-controlled clinical trial. J. Acad. Nutr. Dietetics 115 (3), 369–377. 10.1016/j.jand.2014.11.001 25578927

[B50] Joseph S. V. Edirisinghe I. Burton-Freeman B. M. (2014). Berries: anti-inflammatory effects in humans. J. Agric. Food Chem. 62 (18), 3886–3903. 10.1021/jf4044056 24512603

[B51] Joseph S. V. Edirisinghe I. Burton-Freeman B. M. (2016). Fruit polyphenols: a review of anti-inflammatory effects in humans. Crit. Rev. Food Sci. Nutr. 56 (3), 419–444. 10.1080/10408398.2013.767221 25616409

[B52] Juranić Z. Žižak Ž. (2005). Biological activities of berries: from antioxidant capacity to anti-cancer effects. Biofactors 23 (4), 207–211. 10.1002/biof.5520230405 16498207

[B53] Kalidindi S. (2014). Potential neuroprotective effects of blueberry and lingonberry fruits and leaves. Dr. Diss. Meml. Univ. Nfld.

[B54] Kang J. Thakali K. M. Jensen G. S. Wu X. (2015). Phenolic acids of the two major blueberry species in the US Market and their antioxidant and anti-inflammatory activities. Plant Foods Hum. Nutr. 70 (1), 56–62. 10.1007/s11130-014-0461-6 25535004

[B55] Karlsen A. Paur I. Bøhn S. K. Sakhi A. K. Borge G. I. Serafini M. (2010). Bilberry juice modulates plasma concentration of NF-kappaB related inflammatory markers in subjects at increased risk of CVD. Eur. J. Nutr. 49, 345–355. 10.1007/s00394-010-0092-0 20119859

[B56] Katsube N. Iwashita K. Tsushida T. Yamaki K. Kobori M. (2003). Induction of apoptosis in cancer cells by bilberry (*Vaccinium myrtillus*) and the anthocyanins. J. Agric. Food Chem. 51, 68–75. 10.1021/jf025781x 12502387

[B57] Kaume L. Howard L. R. Devareddy L. (2012). The blackberry fruit: a review on its composition and chemistry, metabolism and bioavailability, and health benefits. J. Agric. Food Chem. 60 (23), 5716–5727. 10.1021/jf203318p 22082199

[B58] Kay C. D. Holub B. J. (2002). The effect of wild blueberry (*Vaccinium angustifolium*) consumption on postprandial serum antioxidant status in human subjects. Br. J. Nutr. 88 (4), 389–398. 10.1079/BJN2002665 12323088

[B59] Keane K. M. Bell P. G. Lodge J. K. Constantinou C. L. Jenkinson S. E. Bass R. (2016). Phytochemical uptake following human consumption of Montmorency tart cherry (*Prunus cerasus* L.) and influence of phenolic acids on vascular smooth muscle cells *in vitro* . Eur. J. Nutr. 55, 1695–1705. 10.1007/s00394-015-0988-9 26163338

[B60] Klavins L. Klavina L. Huna A. Klavins M. (2015). Polyphenols, carbohydrates and lipids in berries of Vaccinium species. Environ. Exp. Biol. 13, 147–158.

[B61] Kolehmainen M. Mykkänen O. Kirjavainen P. V. Leppänen T. Moilanen E. Adriaens M. (2012). Bilberries reduce low‐grade inflammation in individuals with features of metabolic syndrome. Mol. Nutr. and Food Res. 56 (10), 1501–1510. 10.1002/mnfr.201200195 22961907

[B62] Kranz S. Guellmar A. Olschowsky P. Tonndorf-Martini S. Heyder M. Pfister W. (2020). Antimicrobial effect of natural berry juices on common oral pathogenic bacteria. Antibiotics 9 (9), 533. 10.3390/antibiotics9090533 32847029 PMC7557983

[B63] Krentz A. J. (2003). Lipoprotein abnormalities and their consequences for patients with type 2 diabetes. Diabetes,Oobesity and Metabolism 5, S19–S27. 10.1046/j.1462-8902.2003.0310.x 14984018

[B64] Kresty L. A. Frankel W. L. Hammond C. D. Baird M. E. Mele J. M. Stoner G. D. (2006). Transitioning from preclinical to clinical chemopreventive assessments of lyophilized black raspberries: interim results show berries modulate markers of oxidative stress in Barrett's esophagus patients. Nutr. Cancer 54 (1), 148–156. 10.1207/s15327914nc5401_15 16800781

[B65] Krikorian R. Shidler M. D. Nash T. A. Kalt W. Vinqvist-Tymchuk M. R. Shukitt-Hale B. (2010). Blueberry supplementation improves memory in older adults. J. Agric. Food Chem. 58 (7), 3996–4000. 10.1021/jf9029332 20047325 PMC2850944

[B66] Kryvtsova M. Salamon I. Koscova J. Spivak M. (2020). Antibiofilm forming, antimicrobial activity and some biochemical properties of *Vaccinium vitis-idaea* leaf and berry extracts on *Staphylococcus aureus* . Biosyst. Divers. 28 (3), 238–242. 10.15421/012031

[B67] Kylli P. (2010). Berry phenolics: isolation, analysis, identification, and antioxidant properties. Available at: http://urn.fi/URN:ISBN:978-952-10-7115-7.

[B68] Land Lail H. Feresin R. G. Hicks D. Stone B. Price E. Wanders D. (2021). Berries as a treatment for obesity-induced inflammation: evidence from preclinical models. Nutrients 13 (2), 334. 10.3390/nu13020334 33498671 PMC7912458

[B70] Lee I. Chan Y. Lin C. Lee W. Sheu W. H. H. (2008). Effect of cranberry extracts on lipid profiles in subjects with Type 2 diabetes. Diabet. Med. 25 (12), 1473–1477. 10.1111/j.1464-5491.2008.02588.x 19046248

[B71] Lee S. G. Kim B. Yang Y. Pham T. X. Park Y.-K. Manatou J. (2014). Berry anthocyanins suppress the expression and secretion of proinflammatory mediators in macrophages by inhibiting nuclear translocation of NF-κB independent of NRF2-mediated mechanism. J. Nutr. Biochem. 25 (4), 404–411. 10.1016/j.jnutbio.2013.12.001 24565673

[B72] Lehtonen H.-M. Suomela J. Tahvonen R. Yang B. Venojärvi M. Viikari J. (2011). Different berries and berry fractions have various but slightly positive effects on the associated variables of metabolic diseases on overweight and obese women. Eur. J. Clin. Nutr. 65 (3), 394–401. 10.1038/ejcn.2010.268 21224867

[B73] Lewis E. D. Ren Z. DeFuria J. Obin M. S. Meydani S. N. Wu D. (2018). Dietary supplementation with blueberry partially restores T-cell-mediated function in high-fat-diet-induced obese mice. Br. J. Nutr. 119 (12), 1393–1399. 10.1017/S0007114518001034 29845904

[B74] Li D. Zhang Y. Liu Y. Sun R. Xia M. (2015). Purified anthocyanin supplementation reduces dyslipidemia, enhances antioxidant capacity, and prevents insulin resistance in diabetic patients. J. Nutr. 145 (4), 742–748. 10.3945/jn.114.205674 25833778

[B75] Lim D. W. Lee J. E. Lee C. Kim Y. T. (2024). Natural products and their neuroprotective effects in degenerative brain diseases: a comprehensive review. Int. J. Mol. Sci. 25 (20), 11223. 10.3390/ijms252011223 39457003 PMC11508681

[B76] Liu X. m. Liu Y. j. Huang Y. Yu H. j. Yuan S. Tang B. w. (2017). Dietary total flavonoids intake and risk of mortality from all causes and cardiovascular disease in the general population: a systematic review and meta‐analysis of cohort studies. Mol. Nutr. and Food Res. 61 (6), 1601003. 10.1002/mnfr.201601003 28054441

[B77] Lozovoy M. A. B. Oliveira S. R. Venturini D. Morimoto H. K. Miglioranza L. H. S. Dichi I. (2013). Reduced-energy cranberry juice increases folic acid and adiponectin and reduces homocysteine and oxidative stress in patients with the metabolic syndrome. Br. J. Nutr. 110 (10), 1885–1894. 10.1017/S0007114513001207 23750500

[B78] Määttä-Riihinen K. R. Kamal-Eldin A. Mattila P. H. González-Paramás A. M. Törrönen A. R. (2004a). Distribution and contents of phenolic compounds in eighteen scandinavian berry species. J. Agric. Food Chem. 52 (14), 4477–4486. 10.1021/jf049595y 15237955

[B79] Määttä-Riihinen K. R. Kamal-Eldin A. Törrönen A. R. (2004b). Identification and quantification of phenolic compounds in berries of fragaria and rubus species (family rosaceae). J. Agric. Food Chem. 52 (20), 6178–6187. 10.1021/jf049450r 15453684

[B80] Manganaris G. A. Goulas V. Vicente A. R. Terry L. A. (2014). Berry antioxidants: small fruits providing large benefits. J. Sci. Food Agric. 94 (5), 825–833. 10.1002/jsfa.6432 24122646

[B81] Martineau L. C. Couture A. Spoor D. Benhaddou-Andaloussi A. Harris C. Meddah B. (2006). Anti-diabetic properties of the Canadian lowbush blueberry *Vaccinium angustifolium* Ait. Phytomedicine 13 (9-10), 612–623. 10.1016/j.phymed.2006.08.005 16979328

[B82] Mattila P. Hellström J. Törrönen R. (2006). Phenolic acids in berries, fruits, and beverages. J. Agric. Food Chem. 54 (19), 7193–7199. 10.1021/jf0615247 16968082

[B83] Maya-Cano D. A. Arango-Varela S. Santa-Gonzalez G. A. (2021). Phenolic compounds of blueberries (Vaccinium spp) as a protective strategy against skin cell damage induced by ROS: a review of antioxidant potential and antiproliferative capacity. Heliyon 7 (2), e06297. 10.1016/j.heliyon.2021.e06297 33665449 PMC7903303

[B84] Mazza G. Kay C. D. Cottrell T. Holub B. J. (2002). Absorption of anthocyanins from blueberries and serum antioxidant status in human subjects. J. Agric. Food Chem. 50 (26), 7731–7737. 10.1021/jf020690l 12475297

[B85] McCullough M. L. Peterson J. J. Patel R. Jacques P. F. Shah R. Dwyer J. T. (2012). Flavonoid intake and cardiovascular disease mortality in a prospective cohort of US adults. Am. J. Clin. Nutr. 95 (2), 454–464. 10.3945/ajcn.111.016634 22218162 PMC3260072

[B86] Meireles M. Marques. C. Norberto S. Santos P. Fernandes I. Mateus N. (2016). Anthocyanin effects on microglia M1/M2 phenotype: consequence on neuronal fractalkine expression. Behav. Brain Res. 305, 223–228. 10.1016/j.bbr.2016.03.010 26965567

[B87] Mink P. J. Scrafford C. G. Barraj L. M. Harnack L. Hong C.-P. Nettleton J. A. (2007). Flavonoid intake and cardiovascular disease mortality: a prospective study in postmenopausal women. Am. J. Clin. Nutr. 85 (3), 895–909. 10.1093/ajcn/85.3.895 17344514

[B88] Mykkänen O. T. Huotari A. Herzig K.-H. Dunlop T. W. Mykkänen H. Kirjavainen P. V. (2014). Wild blueberries (Vaccinium myrtillus) alleviate inflammation and hypertension associated with developing obesity in mice fed with a high-fat diet. PLoS One 9 (12), e114790. 10.1371/journal.pone.0114790 25501421 PMC4264776

[B89] Nemzer B. V. Al-Taher F. Yashin A. Revelsky I. Yashin Y. (2022). Cranberry: chemical composition, antioxidant activity and impact on human health: overview. Molecules 27 (5), 1503. 10.3390/molecules27051503 35268605 PMC8911768

[B90] Nile S. H. Park S. W. (2014). Edible berries: bioactive components and their effect on human health. Nutrition 30 (2), 134–144. 10.1016/j.nut.2013.04.007 24012283

[B91] Norberto S. Silva S. Meireles M. Faria A. Pintado M. Calhau C. (2013). Blueberry anthocyanins in health promotion: a metabolic overview. J. Funct. Foods 5 (4), 1518–1528. 10.1016/j.jff.2013.08.015

[B92] O’Keefe J. H. Gheewala N. M. O’Keefe J. O. (2008). Dietary strategies for improving post-prandial glucose, lipids, inflammation, and cardiovascular health. J. Am. Coll. Cardiol. 51 (3), 249–255. 10.1016/j.jacc.2007.10.016 18206731

[B93] Olas B. (2018). Berry phenolic antioxidants–implications for human health? Front. Pharmacol. 9, 78. 10.3389/fphar.2018.00078 29662448 PMC5890122

[B94] Olas B. Kontek B. Malinowska P. Żuchowski J. Stochmal A. (2016). *Hippophae rhamnoides* L. fruits reduce the oxidative stress in human blood platelets and plasma. Oxidative Med. Cell. Longev. 2016 (1), 4692486. 10.1155/2016/4692486 PMC4737000 26933473

[B95] Olsson M. E. Gustavsson K. E. Andersson S. Nilsson A. Duan R. D. (2004). Inhibition of cancer cell proliferation *in vitro* by fruit and berry extracts and correlations with antioxidant levels. J. Agric. Food Chem. 52, 7264–7271. 10.1021/jf030479p 15563205

[B96] Pandey K. B. Rizvi S. I. (2009). Plant polyphenols as dietary antioxidants in human health and disease. Oxid. Med. Cell. Longev. 2, 270–278. 10.4161/oxim.2.5.9498 20716914 PMC2835915

[B97] Pap N. Fidelis M. Azevedo L. do Carmo M. A. V. Wang D. Mocan A. (2021). Berry polyphenols and human health: evidence of antioxidant, anti-inflammatory, microbiota modulation, and cell-protecting effects. Curr. Opin. Food Sci. 42, 167–186. 10.1016/j.cofs.2021.06.003

[B98] Paredes-López O. Cervantes-Ceja M. L. Vigna-Pérez M. Hernández-Pérez T. (2010). Berries: improving human health and healthy aging, and promoting quality life—a review. Plant Foods Hum. Nutr. 65, 299–308. 10.1007/s11130-010-0177-1 20645129

[B99] Pedersen C. B. Kyle J. Jenkinson A. Gardner P. McPhail D. Duthie G. (2000). Effects of blueberry and cranberry juice consumption on the plasma antioxidant capacity of healthy female volunteers. Eur. J. Clin. Nutr. 54 (5), 405–408. 10.1038/sj.ejcn.1600972 10822287

[B100] Pojer E. Mattivi F. Johnson D. Stockley C. S. (2013). The case for anthocyanin consumption to promote human health: a review. Compr. Rev. Food Sci. Food Saf. 12 (5), 483–508. 10.1111/1541-4337.12024 33412667

[B101] Puupponen-Pimia R. Aura A.-M. Karppinen S. Oksman-Caldentey K.-M. Poutanen K. (2004). Interactions between plant bioactive food ingredients and intestinal flora—effects on human health. Biosci. Microflora 23 (2), 67–80. 10.12938/bifidus.23.67

[B102] Rahman K. (2007). Studies on free radicals, antioxidants, and co-factors. Clin. Interventions Aging 2 (2), 219–236. PMC2684512 18044138

[B103] Retamales J. Hancock J. (2018). Blueberry taxonomy and breeding. Blueberries. 2, 18–60. 10.1079/9781780647265.0018

[B104] Riso P. Klimis-Zacas D. Del Bo’ C. Martini D. Campolo J. Vendrame S. (2013). Effect of a wild blueberry (*Vaccinium angustifolium*) drink intervention on markers of oxidative stress, inflammation and endothelial function in humans with cardiovascular risk factors. Eur. J. Nutr. 52, 949–961. 10.1007/s00394-012-0402-9 22733001

[B105] Rissanen T. H. Voutilainen S. Virtanen J. K. Venho B. Vanharanta M. Mursu J. (2003). Low intake of fruits, berries and vegetables is associated with excess mortality in men: the Kuopio Ischaemic Heart Disease Risk Factor (KIHD) Study. J. Nutr. 133 (1), 199–204. 10.1093/jn/133.1.199 12514290

[B106] Rocha B. S. Nunes C. Pereira C. Barbosa R. M. Laranjinha J. (2014). A shortcut to wide-ranging biological actions of dietary polyphenols: modulation of the nitrate–nitrite–nitric oxide pathway in the gut. Food and Funct. 5, 1646–1652. 10.1039/C4FO00124A 24912104

[B108] Rodriguez-Mateos A. Heiss C. Borges G. Crozier A. (2014a). Berry (poly) phenols and cardiovascular health. J. Agric. Food Chem. 62 (18), 3842–3851. 10.1021/jf403757g 24059851

[B109] Rodriguez-Mateos A. Pino-García R. D. George T. W. Vidal-Diez A. Heiss C. Spencer J. P. E. (2014b). Impact of processing on the bioavailability and vascular effects of blueberry (poly)phenols. Mol. Nutr. and Food Res. 58 (10), 1952–1961. 10.1002/mnfr.201400231 25044909

[B110] Rodriguez-Mateos A. Rendeiro C. Bergillos-Meca T. Tabatabaee S. George T. W. Heiss C. (2013). Intake and time dependence of blueberry flavonoid–induced improvements in vascular function: a randomized, controlled, double-blind, crossover intervention study with mechanistic insights into biological activity. Am. J. Clin. Nutr. 98 (5), 1179–1191. 10.3945/ajcn.113.066639 24004888

[B111] Rodriguez-Mateos A. Vauzour D. Krueger C. G. Shanmuganayagam D. Reed J. Calani L. (2014c). Bioavailability, bioactivity and impact on health of dietary flavonoids and related compounds: an update. Archives Toxicol. 88 (10), 1803–1853. 10.1007/s00204-014-1330-7 25182418

[B112] Ruel G. Lapointe A. Pomerleau S. Couture P. Lemieux S. Lamarche B. (2013). Evidence that cranberry juice may improve augmentation index in overweight men. Nutr. Res. 33 (1), 41–49. 10.1016/j.nutres.2012.11.002 23351409

[B113] Ruel G. Pomerleau S. Couture P. Lamarche B. Couillard C. (2005). Changes in plasma antioxidant capacity and oxidized low-density lipoprotein levels in men after short-term cranberry juice consumption. Metabolism 54 (7), 856–861. 10.1016/j.metabol.2005.01.031 15988692

[B114] Ruel G. Pomerleau S. Couture P. Lemieux S. Lamarche B. Couillard C. (2006). Favourable impact of low-calorie cranberry juice consumption on plasma HDL-cholesterol concentrations in men. Br. J. Nutr. 96 (2), 357–364. 10.1079/BJN20061814 16923231

[B115] Ruel G. Pomerleau S. Couture P. Lemieux S. Lamarche B. Couillard C. (2008). Low-calorie cranberry juice supplementation reduces plasma oxidized LDL and cell adhesion molecule concentrations in men. Br. J. Nutr. 99 (2), 352–359. 10.1017/S0007114507811986 17761017

[B116] Ryu K. J. Park S.-M. Park S. H. Kim I. K. Han H. Kim H. J. (2019). p38 stabilizes snail by suppressing DYRK2-mediated phosphorylation that is required for gsk3β-βTrCP-induced snail degradation. Cancer Res. 79 (16), 4135–4148. 10.1158/0008-5472.CAN-19-0049 31209060

[B117] Schell J. Betts N. M. Foster M. Scofield R. H. Basu A. (2017). Cranberries improve postprandial glucose excursions in type 2 diabetes. Food and Funct. 8 (9), 3083–3090. 10.1039/C7FO00900C PMC9473326 28748974

[B118] Schreckinger M. E. Wang J. Yousef G. Lila M. A. Gonzalez de Mejia E. (2010). Antioxidant capacity and *in vitro* inhibition of adipogenesis and inflammation by phenolic extracts of *Vaccinium floribundum* and *Aristotelia chilensis* . J. Agric. Food Chem. 58 (16), 8966–8976. 10.1021/jf100975m 23654232

[B119] Seeram N. P. (2008). Berry fruits: compositional elements, biochemical activities, and the impact of their intake on human health, performance, and disease. ACS Publ. 56 (3), 627–629. 10.1021/jf071988k 18211023

[B120] Sezer E. D. Oktay L. M. Karadadaş E. Memmedov H. Selvi Gunel N. Sözmen E. (2019). Assessing anticancer potential of blueberry flavonoids, quercetin, kaempferol, and gentisic acid, through oxidative stress and apoptosis parameters on HCT-116 cells. J. Med. Food 22 (11), 1118–1126. 10.1089/jmf.2019.0098 31241392

[B121] Shidfar F. Heydari I. Hajimiresmaiel S. J. Hosseini S. Shidfar S. Amiri F. (2012). The effects of cranberry juice on serum glucose, apoB, apoA-I, Lp(a), and Paraoxonase-1 activity in type 2 diabetic male patients. J. Res. Med. Sci. 17 (4), 355–360. 23267397 PMC3526129

[B122] Singh J. Basu P. S. (2012). Non-nutritive bioactive compounds in pulses and their impact on human health: an overview. Food Nutr. Sci. 3 (12), 1664–1672. 10.4236/fns.2012.312218

[B123] Skrovankova S. Sumczynski D. Mlcek J. Jurikova T. Sochor J. (2015). Bioactive compounds and antioxidant activity in different types of berries. Int. J. Mol. Sci. 16 (10), 24673–24706. 10.3390/ijms161024673 26501271 PMC4632771

[B124] Skyler J. S. Bakris G. L. Bonifacio E. Darsow T. Eckel R. H. Groop L. (2017). Differentiation of diabetes by pathophysiology, natural history, and prognosis. Diabetes 66 (2), 241–255. 10.2337/db16-0806 27980006 PMC5384660

[B125] Sosa V. Moliné T. Somoza R. Paciucci R. Kondoh H. Lleonart M. E. (2013). Oxidative stress and cancer: an overview. Ageing Res. Rev. 12 (1), 376–390. 10.1016/j.arr.2012.10.004 23123177

[B126] Ștefanescu B.-E. Călinoiu L. F. Ranga F. Fetea F. Mocan A. Vodnar D. C. (2020). The chemical and biological profiles of leaves from commercial blueberry varieties. Plants 9 (9), 1193. 10.3390/plants9091193 32932659 PMC7569947

[B127] Stull A. J. Cash K. C. Champagne C. M. Gupta A. K. Boston R. Beyl R. A. (2015). Blueberries improve endothelial function, but not blood pressure, in adults with metabolic syndrome: a randomized, double-blind, placebo-controlled clinical trial. Nutrients 7 (6), 4107–4123. 10.3390/nu7064107 26024297 PMC4488775

[B128] Stull A. J. Cash K. C. Johnson W. D. Champagne C. M. Cefalu W. T. (2010). Bioactives in blueberries improve insulin sensitivity in obese, insulin-resistant men and women. J. Nutr. 140 (10), 1764–1768. 10.3945/jn.110.125336 20724487 PMC3139238

[B129] Sun J. Chu Y. F. Wu X. Liu R. H. (2002). Antioxidant and antiproliferative activities of common fruits. J. Agric. food Chem. 50 (25), 7449–7454. 10.1021/jf0207530 12452674

[B130] Sun J. Liu R. H. (2006). Cranberry phytochemical extracts induce cell cycle arrest and apoptosis in human MCF-7 breast cancer cells. Cancer Lett. 241 (1), 124–134. 10.1016/j.canlet.2005.10.027 16377076

[B131] Szajdek A. Borowska E. (2008). Bioactive compounds and health-promoting properties of berry fruits: a review. Plant Foods Hum. Nutr. 63, 147–156. 10.1007/s11130-008-0097-5 18931913

[B132] Takikawa M. Inoue S. Horio F. Tsuda T. (2010). Dietary anthocyanin-rich bilberry extract ameliorates hyperglycemia and insulin sensitivity via activation of AMP-activated protein kinase in diabetic mice. J. Nutr. 140 (3), 527–533. 10.3945/jn.109.118216 20089785

[B133] Taruscio T. G. Barney D. L. Exon J. (2004). Content and profile of flavanoid and phenolic acid compounds in conjunction with the antioxidant capacity for a variety of northwest Vaccinium berries. J. Agric. Food Chem. 52 (10), 3169–3176. 10.1021/jf0307595 15137871

[B134] Tian Y. Puganen A. Alakomi H. L. Uusitupa A. Saarela M. Yang B. (2018). Antioxidative and antibacterial activities of aqueous ethanol extracts of berries, leaves, and branches of berry plants. Food Res. Int. 106, 291–303. 10.1016/j.foodres.2017.12.071 29579930

[B135] Torronen R. Kolehmainen M. Sarkkinen E. Mykkanen H. Niskanen L. (2012). Postprandial glucose, insulin, and free fatty acid responses to sucrose consumed with blackcurrants and lingonberries in healthy women. Am. J. Clin. Nutr. 96 (3), 527–533. 10.3945/ajcn.112.042184 22854401

[B136] Uttara B. Singh A. V. Zamboni P. Mahajan R. (2009). Oxidative stress and neurodegenerative diseases: a review of upstream and downstream antioxidant therapeutic options. Curr. Neuropharmacol. 7 (1), 65–74. 10.2174/157015909787602823 19721819 PMC2724665

[B137] Vilkickyte G. Raudone L. Petrikaite V. (2020). Phenolic fractions from *Vaccinium vitis-idaea* L. and their antioxidant and anticancer activities assessment. Antioxidants 9 (12), 1261. 10.3390/antiox9121261 33322638 PMC7763140

[B138] Vyas P. Debnath S. C. Igamberdiev A. (2013). Metabolism of glutathione and ascorbate in lingonberry cultivars during *in vitro* and *ex vitro* propagation. Biol. Plant. 57 (4), 603–612. 10.1007/s10535-013-0339-8

[B139] Wallace T. C. (2011). Anthocyanins in cardiovascular disease. Adv. Nutr. 2, 1–7. 10.3945/an.110.000042 22211184 PMC3042791

[B140] Wang N. Strasburg C. Booren G. Booren A. Gray J. (1999). Antioxidant and antiinflammatory activities of anthocyanins and their aglycon, cyanidin, from tart cherries. J. Nat. Prod. 62, 802. 10.1021/np990184z 10347382

[B141] Wang Y. Chang C. F. Chou J. Chen H.-L. Deng X. Harvey B. K. (2005). Dietary supplementation with blueberries, spinach, or spirulina reduces ischemic brain damage. Exp. Neurol. 193 (1), 75–84. 10.1016/j.expneurol.2004.12.014 15817266

[B142] Wilms L. C. Hollman P. C. Boots A. W. Kleinjans J. C. (2005). Protection by quercetin and quercetin-rich fruit juice against induction of oxidative DNA damage and formation of BPDE-DNA adducts in human lymphocytes. Mutat. Research/Genetic Toxicol. Environ. Mutagen. 582 (1-2), 155–162. 10.1016/j.mrgentox.2005.01.006 15781220

[B143] Wilson T. Luebke J. L. Morcomb E. F. Carrell E. J. Leveranz M. C. Kobs L. (2010). Glycemic responses to sweetened dried and raw cranberries in humans with type 2 diabetes. J. Food Sci. 75 (8), H218–H223. 10.1111/j.1750-3841.2010.01800.x 21535498

[B144] Wilson T. Meyers S. Singh A. Limburg P. Vorsa N. (2008). Favorable glycemic response of type 2 diabetics to low‐calorie cranberry juice. J. Food Sci. 73 (9), H241–H245. 10.1111/j.1750-3841.2008.00964.x 19021808

[B145] Zafra‐Stone S. Yasmin T. Bagchi M. Chatterjee A. Vinson J. A. Bagchi D. (2007). Berry anthocyanins as novel antioxidants in human health and disease prevention. Mol. Nutr. Food Res. 51 (6), 675–683. 10.1002/mnfr.200700002 17533652

[B146] Zhang G. Dai X. (2022). Antiaging effect of anthocyanin extracts from bilberry on natural or UV-treated male *Drosophila melanogaster* . Curr. Res. Food Sci. 5, 1640–1648. 10.1016/j.crfs.2022.09.015 36187878 PMC9516408

[B147] Zheng W. Wang S. Y. (2003). Oxygen radical absorbing capacity of phenolics in blueberries, cranberries, chokeberries, and lingonberries. J. Agric. Food Chem. 51 (2), 502–509. 10.1021/jf020728u 12517117

